# Editorial: Entropy in Networked Control

**DOI:** 10.3390/e21040392

**Published:** 2019-04-11

**Authors:** Christoph Kawan

**Affiliations:** Fakultät für Informatik und Mathematik, Universität Passau, Innstraße 33, 94032 Passau, Germany; christoph.kawan@uni-passau.de

**Keywords:** networked control, information-based control, control entropy

## Abstract

This is an editorial article summarizing the scope and contents of the Special Issue *Entropy in Networked Control*.

Networked control systems are spatially distributed systems in which the communication between sensors, controllers and actuators is accomplished through a shared digital communication network. Examples can be found, e.g., in vehicle tracking, underwater communications for remotely controlled surveillance and rescue submarines, remote surgery, space exploration and aircraft design. Another large field of application is in modern industrial systems, where industrial production is combined with information and communication technology (“Industry 4.0”). In networked control systems, the analog system outputs must be encoded in finite bit strings to be transmitted over the communication network. Realistic models of networked systems, therefore, challenge the standard assumption of control theory that controllers and actuators have access to continuous-valued state information, i.e., information of infinite precision. Additional difficulties in modeling and analysis arise from issues such as time-delayed communication, lossy communication due to noise or packet dropouts and the need of event-based control strategies. Altogether, the resulting nontrivial interaction between the underlying dynamical system and the communication network necessitates the development of new tools and different approaches that combine ideas from various research fields including information theory, nonlinear dynamical systems, graph theory and classical control theory.

To characterize the properties of a communication network that enable a system operating over this network to function properly, various notions of “control entropy” have been introduced and studied. At the same time, the classical notion of Shannon entropy naturally appears in the analysis of noisy communication channels and, as such, plays a major role in the theory of networked control systems. For further background and detailed accounts on previous work in this field, we refer the reader to the monographs [1,2,3,4] and the survey papers [5,6,7].

This Special Issue contains four contributions that either present new ideas and results or provide overviews and discussions of the existing results in different branches of networked control systems.

The review article [8] by Cetinkaya, Ishii and Hayakawa deals with the important research field of security analysis for networked control systems. As many control systems used in industrial applications rely on communication technologies, in particular, wireless networks and the Internet, they are vulnerable as cyber-attackers may try to corrupt their proper functioning by disrupting the communication network. One particular form of attack, the so-called *Denial-of-Service attack*, prevents the delivery of control and measurement data packets within the communication network of the system. The authors provide an overview of the different mathematical models of such attacks and the associated security analysis approaches studied in the literature for feedback control, state estimation and consensus problems. The focus is on packet dropout attacks by malicious nodes in multi-hop networks and jamming attacks in wireless channels. The paper also provides a discussion of the utility of the models studied in the literature for analyzing the security of existing systems as well as for the development of new attack-resilient control and communication techniques.

The discussion paper by Delvenne [9] proposes the use of category theory to formulate concepts and problems in networked control systems. Delvenne first demonstrates that classical concepts and results in ergodic theory and topological dynamics can be formulated elegantly via categorical constructions. In particular, Kolmogorov–Sinai entropy can be defined via Sinai’s factor theorem as a functor from the category **Erg** of measure-preserving dynamical systems on probability spaces to the category whose objects are the extended nonnegative reals [0,∞] and whose morphisms are induced by the natural order on this set. Similar constructions have been studied before by Gromov [10]. In order to say something about networked control systems, the author proposes an extension of this construction to the larger category of *stochastic behaviors* **StoBeh** that is defined as follows: an object in **StoBeh** is a triple (X,f,M), where *X* is a measurable space, *f* is a measurable transformation on *X* and *M* is a convex set of *f*-invariant probability measures. The morphisms are the factor maps between such dynamical systems that respect the associated sets of probability measures. In the context of (stochastic) control systems, one should think of *X* being the set of all trajectories and *f* being the left shift operator on *X*, cf. also Willems [11]. The entropy functor on **Erg** can now be extended naturally to **StoBeh** by taking the supremum of Kolmogorov–Sinai entropy $h_{\mu }\left(f\right)$ over all measures μ∈M associated with a stochastic behavior (X,f,M). The big advantage of using the category **StoBeh** in the context of networked control systems is that it not only allows the definition of different components of a plant such as the open-loop system, the controller and communication channels in formally the same way but also makes it very easy to define the interconnections of such components by simply taking intersections. Moreover, control problems can be specified by introducing appropriate sets of invariant measures. For instance, in the context of set-invariance for a compact set *K*, one may consider all measures supported on the set of trajectories evolving in *K*. Amongst many other things, it remains to show that the (extended) Kolmogorov–Sinai entropy functor is useful in describing the data-rate limits as typically studied in networked control systems.

The paper by Kawan [12] answers a fundamental question in the context of state estimation over noiseless digital channels for nonlinear systems. It is well-known [13,14] that the smallest data rate above which the state of a plant can be estimated with an arbitrarily small error by an observer, obtaining its information via a digital channel, is given by the topological entropy of the plant. However, the operational utility of topological entropy in the design of coding and estimation schemes is questionable, since this quantity is highly discontinuous as a function of system parameters and is very hard to estimate numerically. Another disadvantage of the existing schemes based on topological entropy is that they necessitate an initial accuracy of the estimate which, in general, needs to be tiny in comparison to the aspired accuracy over arbitrarily long time intervals. A possible remedy for all of these problems is provided in the paper [15] by Matveev and Pogromsky, that introduces a quantity named restoration entropy which measures the smallest data rate above which a more robust form of state estimation can be achieved. In particular, this quantity has the following advantages when compared to topological entropy:

- it overcomes the problem of a drastic degradation of the initial estimation error,
- it allows for an explicit formula in terms of the singular values of the linearized system, and
- it depends upper semicontinuously on system parameters.

The paper [12] shows that restoration entropy, in general, strictly exceeds topological entropy, a fact that was not rigorously proved before. More precisely, the main result demonstrates (for the class of mixing Anosov diffeomorphisms) that the equality of topological and restoration entropy for a given system implies a great amount of uniformity which can be expressed in terms of the unstable Lyapunov exponents at each point, the sum of which has to be constant. Such uniformity can easily be destroyed by a small perturbation. The operational meaning of this result is that robust estimation policies, in general, require a higher rate of data transmission than the non-robust ones, as one would expect.

The paper by Voortman, Pogromsky, Matveev and Nijmeijer [16] also studies the problem of state estimation via a noiseless digital channel for both discrete- and continuous-time nonlinear systems. The main contribution of the paper consists in a new coder and observer design so that the associated data rate can be estimated from above by the product of the upper box dimension of the set of relevant initial states (which is assumed to be compact and invariant under the dynamical system) and the largest singular value of the Jacobian of the system. This is particularly interesting, because, for many prototypical chaotic dynamical systems, the interesting dynamics occur on fractal-like sets the dimension of which is strictly less than the dimension of the full state space. At the same time, the proposed coding and estimation scheme is shown to be robust (to a certain extent) against losses in the transmission of data over the channel, a very useful property in the context of networked control systems. With the help of Lyapunov-like functions (used to optimize the singular value estimate) and the notion of Lyapunov dimension (rather than upper box dimension), constructive estimates of the necessary data rates are provided. Explicit computations based on these estimates are carried out for the Lorenz system and the smoothened Lozi map, both of which are examples of chaotic systems intensively studied in the dynamical systems literature.
